# Oral tolerance induction with heated egg in childrena and adolescents

**DOI:** 10.1186/2045-7022-3-S3-P12

**Published:** 2013-07-25

**Authors:** L Villafana, S Terrados, A Ibrahim, B De la Hoz, P Berges-Gimeno

**Affiliations:** 1 Hospital Ramón y Cajal, Madrid, Spain

## Background

The oral tolerance induction to egg (OTI) is a promising treatment modality for egg allergy. Several small pilot studies have been published using different protocols and only one positive double-blind placebo-controlled food challenge have proved efficacy and relatively good tolerance. In all of them raw egg has been used.

Recent studies suggest that a diet with heated egg in patients allergic to regular egg but tolerant to heated egg induces immunological changes that can accelerate their allergy’s resolution. In addition very recent information in murine models shows that extensively heating egg allergen decreases allergenicity and increases safety while still retaining the ability to induce effective desensitization. The objective of this study is to demonstrate that OTI with cooked egg with is a simple and reproducible method to achieve a desensitization or egg tolerance in the future in a safer, more comfortable and probably more effective way.

## Methods

14 patients from 7 to 14 year-old patients with a positive food challenge with boiled egg were selected for our protocol using a mixture of milk and heated egg with an approximate concentration of 350 mg egg/ml. OTI was performed with weekly increasing doses until the ingestion of one egg in omelette. Then the patients continued with the ingestion of at least 3 eggs weekly.

## Results

All 14 patients completed the protocol. Reactions presented during the process were mild without requiring any treatment in the most of patients and only one of them required adrenaline. Mean duration of OTI was 16 weeks. No one experienced reactions during the maintenance period at home. After 6 months of regular intake of heated egg a decrease of the specific IgE(sIgE) to ovalbumin and ovomucoid was noted but in a significant way only for the ovomucoid-sIgE.

## Conclusion

The OTI with heated egg could be a safe and effective way to achieve desensitization or oral tolerance to egg. Evaluating IgE levels all along the protocol suggest that immunologic changes are similar to those reached with OIT using raw egg.

## Disclosure of interest

None declared.
